# Towards the development of upgrade criteria for the treatment of hidradenitis suppurativa with biologics

**DOI:** 10.1111/jdv.70118

**Published:** 2025-11-13

**Authors:** Georgios Nikolakis, Erkan Alpsoy, Monika Arenbergerova, Falk G. Bechara, Farida Benhadou, Joana Cabete, Raffaele Dante Caposiena Caro, Giovanni Damiani, Maia Delage Toriel, Veronique Del Marmol, Valentina Dini, Evangelos J. Giamarellos‐Bourboulis, Katalin Glasenhardt, Philippe Guillem, Ariela Hafner, Evgeniya Hristakieva, John R. Ingram, Vaiva Jarienè, Gregor B. E. Jemec, Niamh Kearney, Brian Kirby, Natalia Kirsten, Piotr K. Krajewski, Vesta Kucinskiene, Aikaterini I. Liakou, Flavia Manzo Margiotta, Angelo V. Marzano, Antonio Martorell, Lukasz Matusiak, Dillon Mintoff, Alejandro Molina Leyva, Francesca Prignano, Tadas Raudonis, Hans Christian Ring, Marco Romanelli, Samed Şahin, Ditte M. L. Saunte, Linnea Thorlacius, Thrasyvoulos Tzellos, Hessel H. Van Der Zee, Kelsey Van Straalen, Christos C. Zouboulis

**Affiliations:** ^1^ Departments of Dermatology, Venereology, Allergology and Immunology, Städtisches Klinikum Dessau Brandenburg Medical School Theodor Fontane and Faculty of Health Sciences Brandenburg Dessau Germany; ^2^ European Hidradenitis Suppurativa Foundation e.V. Dessau Germany; ^3^ Department of Dermatology and Venereology Akdeniz University Antalya Türkiye; ^4^ Department of Dermatovenerology, Third Faculty of Medicine Charles University and University Hospital Kralovske Vinohrady Prague Czech Republic; ^5^ International Centre for Hidradenitis Suppurativa/Acne Inversa (ICH), Department of Dermatology, Venereology and Allergology Ruhr‐University Bochum Bochum Germany; ^6^ Department of Dermatology Hôpitaux Universitaires de Bruxelles (HUB), Université libre de Bruxelles Brussels Belgium; ^7^ Department of Dermatology and Venereology Hospital de Santo António dos Capuchos – ULS de São José Lisbon Portugal; ^8^ Dermatology Clinic Maggiore Hospital, University of Trieste Trieste Italy; ^9^ Department of Biomedical, Surgical and Dental Sciences University of Milan Milan Italy; ^10^ Italian Center of Precision Medicine and Chronic Inflammation University of Milan Milan Italy; ^11^ L'Oréal et Institut Pasteur Paris France; ^12^ Department of Dermatology Hôpital Erasme – Hôpitaux universitaires de Bruxelles (HUB) Brussels Belgium; ^13^ Dermatology Unit Department of Clinical and Experimental Medicine Ospedale Santa Chiara Pisa Italy; ^14^ 4th Department of Internal Medicine National and Kapodistrian University of Athens, Medical School Athens Greece; ^15^ Department of Dermatology and Allergology University of Szeged Szeged Hungary; ^16^ Clinique du Val d'Ouest, Service de Chirurgie Ecully France; ^17^ Department of Dermatology Tel Aviv Sourasky Medical Center Tel Aviv Israel; ^18^ Clinic of Dermatology and Venereology UMHAT "Prof. Dr. Stoyan Kirkovich" AD Stara Zagora Bulgaria; ^19^ Section of Dermatovenereology, Faculty of Medicine Trakia University Stara Zagora Bulgaria; ^20^ Division of Infection and Immunity Cardiff University Cardiff UK; ^21^ Department of Skin and Venereal Diseases Lithuanian University of Health Sciences (LSMU), Hospital of LSMU Kauno Klinikos Kaunas Lithuania; ^22^ Department of Allergy and Dermatology Herlev & Gentofte University Hospital Gentofte Denmark; ^23^ Department of Clinical Medicine, Faculty of Health and Medical Sciences University of Copenhagen Copenhagen Denmark; ^24^ Department of Dermatology Our Lady of Lourdes Hospital Drogheda Drogheda Ireland; ^25^ School of Medicine University College Dublin Dublin Ireland; ^26^ Department of Dermatology St. Vincent's University Hospital Dublin Dublin Ireland; ^27^ Department of Dermatology and Allergy University Hospital, LMU Munich Munich Germany; ^28^ University Centre of General Dermatology and Oncodermatology Wroclaw Medical University Wroclaw Poland; ^29^ 1st Department of Dermatology‐Venereology, "Andreas Sygros" Hospital, Medical School National and Kapodistrian University of Athens Athens Greece; ^30^ Health Science Interdisciplinary Center Sant'Anna School of Advanced Studies Pisa Italy; ^31^ Dermatology Unit Fondazione IRCCS Ca' Granda Ospedale Maggiore Policlinico Milan Italy; ^32^ Department of Pathophysiology and Transplantation Università degli Studi di Milano Milan Italy; ^33^ Department of Dermatology Hospital de Manises Valencia Spain; ^34^ Faculty of Medicine Wroclaw University of Science and Technology Wroclaw Poland; ^35^ Department of Dermatology Mater Dei Hospital Msida Malta; ^36^ Hospital Universitario Virgen de las Nieves Servicio de Dermatología‐Ibs.Granada Granada Spain; ^37^ Department of Dermatology University of Granada Granada Spain; ^38^ Department of Health Science, Dermatology Section University of Florence Florence Italy; ^39^ Clinic of Infectious Disease and Dermatovenereology Institute of Clinical Medicine, Faculty of Medicine, Vilnius University Vilnius Lithuania; ^40^ Department of Dermatology Zealand University Hospital Roskilde Denmark; ^41^ Department of Dermatology Faculty of Medicine, Gazi University Ankara Turkey; ^42^ Afdelingen for Hud‐ og Kønssygdomme Aarhus Universitetshospital Arahus Denmark; ^43^ Section for Biostatistics and Evidence‐Based Research The Parker Institute, Bispebjerg and Frederiksberg Hospital Copenhagen Denmark; ^44^ Department of Dermatology Nordland Hospital Trust Bodø Norway; ^45^ Department of Dermatology Erasmus Medical Center Rotterdam the Netherlands

**Keywords:** adalimumab, bimekizumab, biologics, hidradenitis suppurativa, secukinumab, upgrade criteria

## Abstract

**Background:**

Hidradenitis suppurativa (HS) is a chronic inflammatory skin disease characterized primarily by dysregulated innate immunity and abnormal keratinocyte differentiation. Over the past two decades, the treatment paradigm has evolved from antibiotics to targeted biologics, such as anti‐TNF‐α and anti‐IL‐17A/A‐F inhibitors. However, antibiotic therapy remains a prerequisite for biologic initiation despite a lack of comparative studies evaluating their efficacy.

**Objectives:**

This study aimed to assess current prescription patterns for systemic HS therapies among European HS specialists, evaluate unmet needs in antibiotic and biologic use and explore expert opinions on criteria for biologic upgrade as a first‐line therapy.

**Methods:**

A structured questionnaire was distributed to 55 HS specialists, the majority of whom were members of the European Hidradenitis Suppurativa Foundation (EHSF), comprising HS experts and future opinion leaders. Responses underwent statistical analysis to assess trends in antibiotic versus biologic prescription, treatment efficacy and potential improvements in therapeutic decision‐making.

**Results:**

A total of 43 participants (76.6% of invited experts, 80% of invited opinion leaders) responded. While 95% adhered to licensing regulations mandating 10–12 weeks of antibiotics before biologics, 81% acknowledged prescribing antibiotics despite anticipating inadequate responses. More than half reported patient‐reported flares during antibiotic treatment. The majority (77%) supported earlier biologic initiation in cases of persistent flares, and 79% favoured short‐term biologic therapy over antibiotics for early‐stage HS. Participants identified specific phenotypic attributes such as rapidly progressing disease, extensive involvement and comorbidities as factors warranting earlier biologic intervention.

**Conclusions:**

Current treatment practice may delay optimal intervention, potentially leading to missed therapeutic windows. A significant proportion of respondents expressed a preference for earlier biologic intervention, especially in cases of severe disease or frequent flares. The findings underscore the need for a consensus statement defining upgrade criteria for biologics as a first‐line therapy, potentially improving patient outcomes and reducing healthcare burdens.

## Plain language summary (PLS)

**What Is the Disease?:**

Hidradenitis suppurativa (HS) is a painful, long‐term skin condition. It causes boils, abscesses and channels under the skin, mostly in areas such as the armpits, groin and buttocks. HS can seriously affect quality of life and is more common than many people may realize.

**Location of the Study:**

This research was conducted across 22 European countries, involving dermatology experts from the European Hidradenitis Suppurativa Foundation (EHSF).

**Aim of the Study:**

The main goal was to find out when and why doctors might choose modern medicines called biologics, rather than traditional antibiotics, for patients with HS.

**Methodology:**

Doctors who are experts in treating HS received a survey about their current use of antibiotics and biologics. A total of 43 specialists participated, representing both very experienced doctors and newer opinion leaders in the field. They were asked about how they treat HS and what situations might call for earlier use of biologics.

**Key Findings:**

- Nearly all doctors still prescribe antibiotics for at least 10–12 weeks before starting biologics, even if they do not expect antibiotics to work well.
- Many patients have ongoing skin flare‐ups while taking antibiotics.
- Most doctors agree that biologics should sometimes be started earlier, especially for patients whose HS is severe, worsening rapidly or not responding to antibiotics.

**Conclusions and Practical Implications:**

Current rules may delay more effective treatment, leading to worse outcomes for patients. The study shows strong support among HS specialists for clear recommendations that allow for earlier use of biologics in certain cases. Adopting these changes could help patients receive better, faster care and prevent progression of the disease.

## Graphical abstract

Forty‐three HS specialists from 22 European countries reported adherence to HS guidelines and use of antibiotics before biologics despite limited efficacy and rapid relapse (49% within 1–3 months). Seventy‐nine per cent supported short‐term biologics for severe Hurley I disease. Further consensus‐building is essential for phenotype‐based upgrade criteria for biologics in HS.

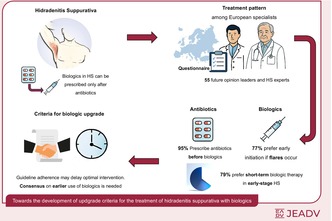

**Why was the study undertaken?**

Phase III HS studies with biologics have traditionally required Hurley II–III patients with irreversible lesions (draining tunnels) for inclusion, overlooking opportunities for earlier intervention. Early aggressive treatment during the optimal ‘window of opportunity’ could prevent progression to irreversible damage.

**What does this study add?**

Nearly half of surveyed participants reported HS patients' flares within 1–3 months after discontinuing prolonged antibiotic therapy. 79% of the participants would prefer short‐term biologics to antibiotics for Hurley I severe disease. A number of different phenotypic constellations were proposed for an upgrade to biologics.

**What are the implications of this study for disease understanding and/or clinical care?**

A significant proportion of respondents expressed a preference for earlier biologic intervention, especially in cases of severe disease or frequent flares, with no significant differences between experts and future opinion leaders. The study provides the rationale for the development of upgrade criteria for the use of biologics in HS. Further consensus‐building is required to provide a proposal for the appropriate phenotypes for first‐line biologic treatment.

## INTRODUCTION

Novel research findings on hidradenitis suppurativa/acne inversa (HS) over the past two decades^1^, ^2^, ^3^ have redefined the disease from a primarily infectious one to a multifactorial inflammatory skin disease with a complex pathogenesis centred on a disruption of the innate immunity and abnormal keratinocyte differentiation of the hair follicle. This shift in understanding led to the evolution of treating the disease with agents targeting specific components of the inflammatory cascade, such as the TNF‐α inhibitor adalimumab, which was licensed over 10 years ago.^4^ A more comprehensive understanding of HS pathophysiology led to further evolution of therapeutic strategies and the addition of the anti‐IL‐17A and anti‐IL17A/F inhibitors secukinumab and bimekizumab to the therapeutic ‘arsenal’ of dermatologists and HS specialists over the last 2 years.^5^, ^6^, ^7^

Moreover, the development of validated scoring systems, both dichotomous and continuous, aims to accurately represent severity changes in both a dynamic and categorical manner. This approach facilitates a more precise capture of the potential available treatments.^8^, ^9^, ^10^, ^11^, ^12^, ^13^

Biologic treatment is currently restricted by licensing regulation, which mandates a 10‐ to 12‐week course of antibiotics prior to initiation. Various antibiotic treatments have shown efficacy in small randomized control trials, case–control studies and case series, using heterogenous scoring semiquantitative or qualitative methods to define treatment response.^14^, ^15^, ^16^, ^17^, ^18^, ^19^, ^20^, ^21^, ^22^, ^23^, ^24^ A recent prospective multicenter European study employed a well‐validated scoring system to assess the efficacy of doxycycline and clindamycin‐rifampicin^25^ as treatment options for HS patients; however, head‐to‐head prospective studies comparing antibiotics with biologics are lacking.

All Phase III biologic studies included Hurley stage II and III patients, making the presence of scarring or draining tunnels necessary for their inclusion. This currently outdated approach stems from an era where irreversible HS lesions were perceived as a hallmark of disease severity rather than the inevitable consequence of the inadequately treated inflammatory component of the disease.^26^

HS has a high socio‐economic burden with a regular need for patients seeking treatment in a hospital emergency department.^27^, ^28^ In addition, inpatient treatment of HS is often associated with significant direct and indirect costs. These costs not only encompass the medical expenses related to surgery themselves but also include the costs of hospitalization, medications and follow‐up care. Indirect costs can further extend to lost wages for the patients during their recovery period and the potential impact on their overall quality of life. The financial burden can be significant for both the healthcare system and the patients, reinforcing the urgent need for more efficient management and treatment strategies with an earlier positive impact on HS patients' quality of life.

Currently, the understanding and management of inflammatory skin diseases is undergoing a significant paradigm shift, with HS emerging as a prime example of a condition where early and aggressive intervention could be critical for effective treatment.^1^, ^29^ HS serves as a model disease demonstrating the importance of an optimal window of opportunity, where timely and robust therapeutic strategies may not only potentially delay but even reverse disease progression.^30^ Several studies support the idea of a more aggressive approach in order to change disease trajectory in the course of the disease: A multicentre observational study on secukinumab demonstrated that a lower therapeutic burden, defined as fewer prior systemic treatments and surgical interventions, was significantly correlated with higher response.^31^ Results from a registry analysis highlighted that consistent biologic use reduced the need for acute interventions, systemic antibiotics and hospitalizations compared to delayed biologics after irreversible damage, demonstrating an inverse correlation between treatment delay and clinical response to biologics.^30^, ^31^

This evolving approach brings to the forefront the question of whether specific cases or phenotypic constellations of HS subtypes might warrant first‐line biologic treatment, providing advantages both for patients' health outcomes and long‐term sustainability for healthcare. The use of antibiotics in patients who are likely to switch to biologics in the near future should be critically evaluated, particularly in view of the increasing antibiotic resistance worldwide.^32^

To address this, we sought insights from selected physicians, the majority of whom have been associated with the European Hidradenitis Suppurativa Foundation (EHSF), who possess specialized knowledge and experience with HS. Our objective was to better define their systemic therapy strategies and identify potential unmet needs in the prescription of antibiotics and biologics in their clinical practice.

## METHODS

An electronic questionnaire was distributed to 55 HS specialists, with the majority of them being also members of the European Hidradenitis Suppurativa Foundation (EHSF) on 20 July 2024 using Google Forms (California, USA), who were expected to complete it within 40 days. Invitations were distributed via email to the addresses provided by members upon joining the European Hidradenitis Suppurativa Foundation (EHSF). To ensure balanced geographical representation, colleagues from all European countries were considered. Participants were classified into two categories: HS experts and future opinion leaders.

HS experts were defined as physicians aged >40 years with extensive clinical experience in the management of HS, frequent peer‐reviewed publications on the disease and/or contributions to national or international clinical guidelines. Many had served as principal investigators in major clinical trials or disease registries. In addition, they often held leadership roles in professional societies, organized dedicated HS workshops and contributed to major educational initiatives aimed at improving HS care.

Future opinion leaders were defined as younger colleagues (typically aged <40 years) actively involved in emerging HS research, with recent first‐ or co‐author publications, poster presentations or documented active participation in national or international congresses related to HS within the preceding 3 years.

The age of the experts and future opinion leaders was presented as mean ± SD, and the statistical analysis was performed using Jamovi Version 2.6.25.0 (Sydney, Australia).

The questionnaire included 16 questions, which attempted to clarify the prescription patterns of systemic therapies for HS, the implementation of the guidelines in the ‘real‐world’ situation, and to identify potential unmet needs in the prescription of biologics (see also supplemental material 2). In a subsequent step, the participants were asked to describe, if applicable, three cases of phenotypes or clinical constellations in which they would prefer to start biologic treatment as monotherapy or in combination with antibiotics without the previously required 3‐month antibiotic course. The results were statistically presented as mean ± SD or median (1st–3rd quartile), and were statistically evaluated using chi‐squared, Mann–Whitney or Student's t‐test, where applicable, after evaluation of data distribution using the Kolmogorov–Smirnov test, using Python version 3.12.4 software (Python Software Foundation, Beaverton, USA).

Differences were considered significant if the p‐value was <0.05. Visualization of the results was performed using Microsoft Excel Version 16.93.1 (Microsoft Corporation, Washington, USA).

## RESULTS

**FIGURE 1:**
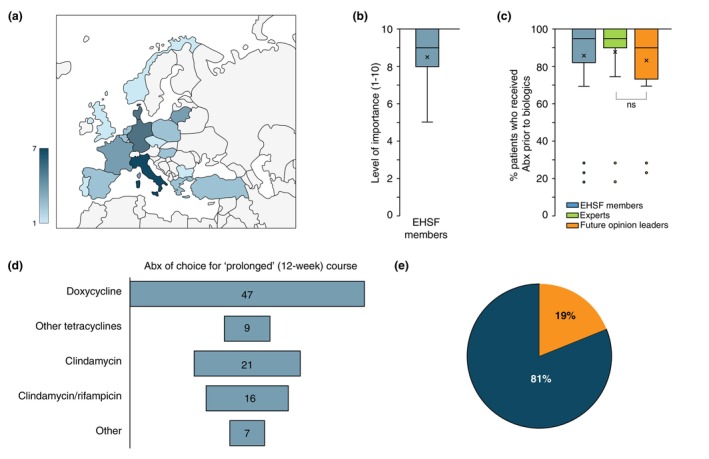
(a) Graphic representation of the 19 out of 22 invited countries, which participated in the survey. The participants from a country varied from 1 to 7 persons. (b) Graph on the level of clinical importance guidelines and consensus statements have for the participants. (c) Box and whisker plot depicting the percentage of patients treated by the participants, experts and opinion leaders, who were pre‐treated with antibiotics before the initiation of biologics. ID: Tree plot demonstrating the most common antibiotics prescribed for the 3‐month course for HS patients from the participants of this study. (e) Percentage of participants who replied yes, admitted they had to prescribe at least once antibiotics as a regulatory necessity in order to initiate biologic treatment, although they did not expect an adequate response.

A total of 23 out of the 30 invited experts (77%) and 20 out of the 25 invited opinion leaders (80%) (total participants = 43) joined the study, with diversified origins from 19 out of 22 invited European countries (Figure 1). 9/23 (39%) of the experts and 11 of 20 (55%) of the future opinion leaders were females. The mean age of the experts was 55.4 ± 7.8 years, while the age of the future opinion leaders was 36.5 ± 4.88 years (*p* < 0.001). The descriptives of the participants are presented in the supplemental material.

Forty‐one of 43 participants (95%) agreed that a consensus statement on the use of biologics as first‐line therapy for HS would be useful for their clinical practice. The participants were asked about the importance of guidelines and consensus agreements for clinical practice, and the median value was 9 out of 10 (8–10), with no significant differences between experts and future opinion leaders (Figure 1b). A total of 81% of the respondents (83% of the experts and 80% of the future opinion leaders) reported prescribing antibiotic therapy as a first‐line treatment due to regulatory requirements, despite not expecting adequate efficacy (Figure 1e).

95% of the specialists indicated that their patients had received biologics as per licensing regulations, with the vast majority (95%) having received prior treatment with antibiotics for 10–12 weeks (Figure 1c). The percentage for future opinion leaders was 90% (74–100) compared to experts (95%, 90–100), but with no statistically significant difference observed.

Doxycycline was the most prescribed antibiotic (47%) (Figure 1d), followed by clindamycin monotherapy (21%), the combination of clindamycin and rifampicin (16%), other tetracycline‐group antibiotics (9%) and lastly other combinations, including agents targeting predominantly Gram‐negative and anaerobic bacteria (7%) (Figure 1d).

**FIGURE 2:**
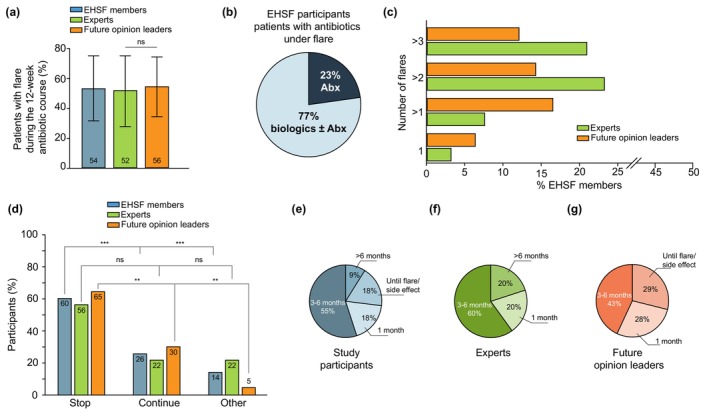
(a) Percentage of participants stating that patient‐reported flares were reported during the 12‐week antibiotic course. n.s: non‐significant. (b) Graph on the therapeutic decision of continuing the antibiotic therapy versus switching to or adding a biologic after a patient reported flare. (c) Number of flares needed according to all the participating EHSF‐members, experts and young opinion leaders in order to initiate biologic treatment before the end of the 12‐week antibiotic course. (d) Graphic depiction of the percentage of participants who would decide to continue the antibiotic treatment or stop it, in case of a responder, n.s: not significant; **P<0.01; ***p<0.001. (e) different time periods of treatment from participants who would decide to continue with the antibiotic therapy after the course of 3 months, both experts (f) and future opinion leaders (g).

Regarding antibiotic efficacy in controlling disease flares, 52 ± 24% of those treated by the expert group and 56 ± 20% of those treated by future opinion leaders were reported to have experienced a flare during the course of the 10‐ to 12‐week antibiotic treatment (Figure 2), with no statistical differences between the different groups (*p* > 0.05). Additionally, 58% of the participants stated that they would then initiate monotherapy with biologics rather than continue with antibiotics, while 74% favoured combining biologics with antibiotics (Figure 2b).

Participants were asked how many patient‐reported flares they would consider significant in order to switch to a biologic agent prior to completing the 12‐week antibiotic course. Most experts required over two flares (23% of the participants) or three flares (21% of the participants) to initiate earlier biologic therapy (Figure 2c), whereas the threshold appeared to be lower (>1 flare) for most of the future opinion leaders (16%), with no statistically significant differences between the two groups (*p* < 0.05).

Another focus of the survey revolved around the treatment strategies concerning patients who responded to antibiotic therapy (Figure 2d). 60% of all the participants chose to discontinue the treatment after 10–12 weeks, while 26% opted to continue the antibiotic therapy (*p* < 0.001). Among experts, 56% would stop the therapy, while 22% supported its continuation (*p* > 0.05). In contrast, 65% of the future opinion leaders discontinued the treatment after the 3‐month course, while 30% preferred continuation of antibiotic therapy (*p* = 0.004). Among those extending antibiotic therapy over 3 months, 18% continued treatment for 1 month, 55% for 4–6 months, 9% for more than 6 months and 18% continued the treatment until a flare or a side effect necessitated discontinuation (Figure 2e). For those continuing antibiotics, 60% of the experts and 43% of the future opinion leaders would prefer to continue the therapy for 3–6 months, while 20% versus 29% would continue the treatment until a flare or a side effect occurred. A short‐term prolongation of the antibiotic course (1 month) was chosen by fewer participants (20% vs. 28%).

Furthermore, participants were asked to estimate the average time to relapse after discontinuing a prolonged course of antibiotic treatment, according to their clinical experience. A total of 49% of them reported a relapse within 1–3 months, 35% within 3–6 months, while 9% highlighted an early disease worsening in less than 1 month.

**FIGURE 3:**
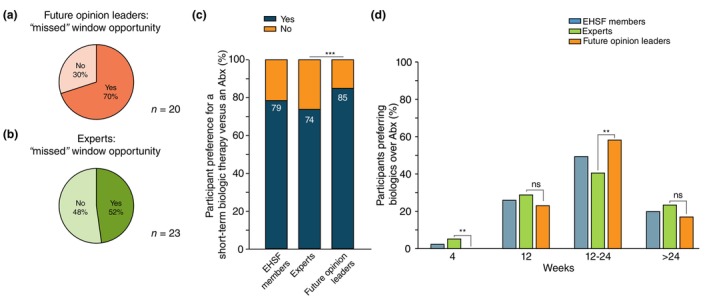
Percentage of experts (a) and future opinion leaders (b) on their view of the theory that the window of opportunity might be missed during the 12‐week antibiotic course. (c) Graphic representation of the percentage of participants who would prefer a short‐term biologic therapy of an antibiotic therapy for Hurley I patients with severe disease, ***p < 0.001. (d) Duration of the ideal biologic treatment period for HS patients with the aforementioned phenotype. n.s: not significant; **p < 0.01.

Participants were asked whether they believed that an antibiotic course lasting over 3 months as per licensing requirements might lead to missing the window of opportunity in HS, leading to Hurley progression and irreversible lesions, such as draining or non‐draining tunnels and cicatrization. A total of 48% of the experts (Figure 3b) and 70% of the young opinion leaders (Figure 3) believed the window of opportunity could be missed, with the difference not being statistically significant (*p* = 0.2).

Lastly, participants were asked to express whether they would prefer a short‐term biologic treatment instead of antibiotic treatment for Hurley I patients (Figure 3c) and, if so, how long this treatment should be (Figure 3d). A total of 79% of participants preferred such treatment, with significantly more future opinion leaders (85%) compared to experts (74%, *p* = 0.006). The preferred duration would be 12 weeks with the option to extend it to 24 weeks (41% vs. 59%, *p* = 0.0019). The second most preferred option was a fixed 12‐week course of biologics.

The majority of responses from both groups concerning specific phenotypic constellations that could be eligible for an upgrade to biologic treatment fell into the following categories: disease severity according to IHS4, number of flares, disease dissemination on both the upper and lower body, fast progression of Hurley stage, disease with concomitant inflammatory comorbidities or syndromic HS phenotypes, specific involvements of the anogenital and/or inguinal region as well as paediatric patients with a family history of HS or patients with inflammatory HS lesions that began in childhood or adolescence (Table 1).

**TABLE 1:** The table summarizes some of the preliminary results of specific clinical scenarios and phenotypic constellations in which early biologic therapy may be considered in HS according to preliminary survey results. These results do not constitute formal guideline recommendations.

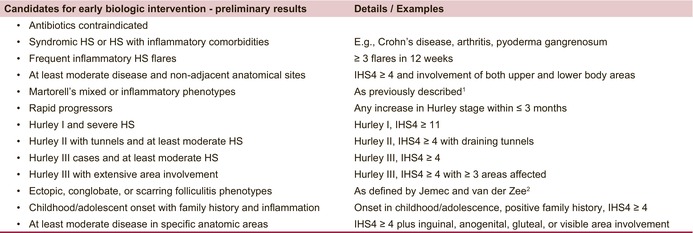

## DISCUSSION

This study aimed to describe an expert opinion on current prescription patterns regarding biologics and antibiotics for HS, to uncover potential unmet needs and to provide the rationale for developing upgrade criteria for the use of biologics as first‐line therapy for HS. Although such recommendations are already in use for other chronic inflammatory skin diseases, for example, psoriasis,^35^, ^36^, ^37^ a clear statement advocating for upgrading HS patients without the need of a 3‐month antibiotic course is not documented clearly in the literature to date. This is surprising, given that the ‘point of no return’ marked by scarring and tunnel formation is more easily identifiable in HS compared to other inflammatory diseases.

In addition, it seems that the treatment of multiple draining tunnels is recognized as the ‘Achilles heel’ of certain modern anti‐inflammatory therapies: A reanalysis of the PIONEER I&II data identified draining tunnels as a major factor influencing clinical response.^38^ Frew et al.^39^ demonstrated through a time‐to‐event analysis that the presence of dermal tunnels significantly increased the time required to achieve HiSCR, with patients needing more than double the time compared to patients without tunnels. A randomized real‐world study compared HS patients treated with adalimumab as monotherapy versus combination with surgery over a course of 12 months. The results highlighted that surgery led to a significantly higher reduction of IHS4 and DLQI, indirectly suggesting that the surgical removal of hard‐to‐treat lesions resulted in a more robust clinical response and better quality of life for HS patients. Draining tunnels might lead to secondary polymicrobial bacterial colonization^40^, ^41^ and there is also evidence for biofilm formation within the lumen of the tunnels which may attribute to recalcitrance towards antibiotics and the prolonged inflammation.^42^, ^43^, ^44^, ^45^ The pathogenesis for such lesions might be more challenging to treat with biologic monotherapy, requiring prolonged antibiotic courses and/or surgical procedures, such as deroofing and local or wide excisions, for adequate disease control. Although anti‐IL‐17 compounds have been shown to have efficacy in treating draining tunnels,^46^ a significant gap remains between the clinical response between Hurley II and III patients in both relevant Phase III trials.^6^, ^7^

The value of a ‘hit hard and early’ approach versus long‐term inflammatory suppression of irreversible lesions was reflected in the responses to this questionnaire. Indeed, participants almost unanimously supported the idea that upgrade criteria for biologic use as first‐line therapy would be beneficial for clinical practice. The respondents showed strong adherence to guidelines, rating their importance at 9 out of 10 and prescribing biologics according to licensing requirements in 95% of cases, following a long course of antibiotics.

HS is known among inflammatory skin diseases for its significant diagnostic delay. A German study estimated this delay at around 10.1 years,^47^ while a more recent French study^48^ conducted after awareness efforts by institutions, patient organizations and the pharmaceutical industry demonstrated a significant reduction in this time to less than half. This underscores the importance of early diagnosis as it impacts the progression of single lesions to draining tunnels. This prospect was highlighted by 70% of future opinion leaders, suggesting that a 3‐month antibiotic course might lead to a missed window of opportunity, while the experts were more divided on this issue, without the difference being significant.

Prospective head‐to‐head studies directly comparing the efficacy of antibiotics versus biologics are lacking, but participants reported that the current prescription pattern, dictated by licensing requirements, cannot adequately capture all HS cases. A total of 81% of responders admitted to prescribing antibiotics without expecting a sufficient response, as it remains a prerequisite for biologic administration. Additionally, 54% of the participants admitted already having patients complaining about flares before the end of the 12‐week antibiotic course. The disease‐free interval is not equally documented for all antibiotic regimens used in HS. For clindamycin‐rifampicin, it is documented to be around 4–5 months.^16^, ^21^ Beyond bacteriostatic or bactericidal effects, antibiotics have also demonstrated purely anti‐inflammatory effects such as inhibiting effects on lymphocyte transformation, antibody production and neutrophile chemotaxis.^49^, ^50^, ^51^, ^52^, ^53^, ^54^ A total of 77% of participants reported initiating biologic treatment earlier, without completing the 12‐week antibiotic course, when flares occurred. Experts primarily required three flares, while young opinion leaders considered two or more flares as an indication of suboptimal disease control, prompting an earlier switch to a biologic agent. The consortium of the German S2k guideline was the first to provide a statement on this matter, proposing a review of the meaningfulness of an extended duration of antibiotic therapy and a possible switch to another therapeutic modality (biologics and/or surgery if necessary) at 3 months the latest.^3^, ^55^

Concerning HS patients who responded to antibiotic therapy, significantly more young opinion leaders compared to experts chose to stop rather than continue antibiotic treatment. Surprisingly, 55% opted to continue the treatment over 3–6 months and 18% persisted until a flare or side effect required discontinuation. For 8 years, adalimumab was the only licensed biologic therapy for HS and the high number of refractory cases, along with the reimbursement challenges across different countries, might explain these results. Despite this, concerns regarding antimicrobial resistance^56^, ^57^ from extensive antibiotic courses and the newly available anti‐IL‐17 compounds might lead to a shift in treatment strategy in the coming years.

A total of 86% of future opinion leaders and 74% of experts favoured a 3‐ to 6‐month biologic treatment course over antibiotics for severe Hurley I patients. This particular phenotype may shape the future of HS treatment, with early diagnosis that subsequently would allow a ‘hit hard and early’ approach for shorter, intensive intervention rather than lifelong immunosuppression of late‐Hurley stage lesions.

A total of 42 out of 43 participants provided several different answers regarding which phenotypic constellations or cases of HS they would deem appropriate for initiating biologic monotherapy as first‐line therapy or for combining biologics and antibiotics, with the initial goal of distinguishing between the two groups. Unfortunately, there was a substantial overlap in the answers of both groups, making this distinction impossible. Many respondents selected similar groupings of clinical features but could not always determine distinctive, group‐specific phenotypes appropriate for early biologic initiation. Thus, while the data reflect general agreement in some clinical scenarios, they lack enough discrimination to support a tailored phenotype‐specific recommendation for upgrading to biologics. Clinical heterogeneity of HS, in combination with the missing sharp demarcation of certain clinical phenotypes, might be possible reasons for the current difficulty in effectively personalizing HS treatment. A possible answer could be a molecular or genotype‐based approach: advances in biomarker discovery, tissue profiling and genome studies might help us identify molecular subgroups of HS patients likely to benefit from early or targeted biologic interventions.

The aforementioned data speak to the need for further clarifications and consensus building. Despite the heterogeneity, answers will be grouped appropriately and will be presented in a next round where a larger number of HS experts and future opinion leaders will be requested to vote on a Likert scale ranging from −5 (strongly disagree) to +5 (strongly agree) on a number of statements during a Delphi consensus conference. We hope that the results of the conference will serve as the foundation for developing an EHSF checklist, including clear recommendations for the upgrade criteria for first‐line biologic therapy in HS.

There are several limitations to our study: despite the considerable amount of participants, the study surveyed a limited number of HS experts and future opinion leaders. This might not appropriately depict the diversity of clinical practice across different European regions and health systems. The distribution of a survey as a method might lead to selection bias, since respondents might have different opinions than non‐respondents. The use of a structured questionnaire narrows the depth of responses and might not capture all clinical considerations or reasoning. Data based on self‐reported opinions and behaviours might be subject to recall bias and not adequately reflect clinical practice. EHSF members may tend to answer in ways they perceive as professionally desirable or aligned with current guidelines rather than reporting true individual beliefs. Although this manuscript is to be perceived as an expert opinion of physicians, lack of the patients' voice, view and perspectives might have added a valuable variable to the interpretation of the results. Lastly, several authors of this manuscript report personal fees, advisory roles and research support from pharmaceutical companies involved in the development of biologic therapies for HS, including participation in related clinical trials. These relationships are detailed in the conflict of interest section and may be relevant to the interpretation of study findings.

## AUTHOR CONTRIBUTIONS

GN: data curation, formal analysis, project administration, writing—original draft preparation: GN, CCZ; conceptualization: TZ, CCZ, FMM; supervision; all authors: data generation, review and editing.

## FUNDING INFORMATION

None.

## CONFLICT OF INTEREST STATEMENT

Georgios Nikolakis has received honoraria and travel grants from UCB, Novartis, Almirall, BMS, AbbVie, Eli Lilly and his institution received honoraria from Mölnlycke GmbH for his participation in advisory boards. Erkan Alpsoy received honoraria from UCB, Abbvie, Lilly and Johnson & Johnson. Falk Bechara has received consulting fees from AbbVie, Moonlake, UCB, Celltrion, Beiersdorf, Novartis, Janssen Cilag, Lilly, Sanofi, Sitala, Incyte, Mölnlycke, Avalo and Acelyrin; honoraria for lectures and support to travel meetings from AbbVie, Boehringer Ingelheim, Celltrion, Dr. Wolff, Janssen Cilag, Mölnlycke, Moonlake, UCB and Novartis; and participated on monitoring and advisory boards from AbbVie, Novartis, Moonlake, UCB, Boehringer Ingelheim and Janssen Cilag. Joana Cabete received consulting fees and travel support for attending meetings from Novartis and is the president of the Portuguese Group of Hidradenitis Suppurativa. Raffaele Dante Caposiena Caro has received honoraria for participation in speaker bureaus from Novartis, Eli Lilly, Sanofi and Pfizer. Valentina Dini has received honoraria from AbbVie, Almirall, Convatec, Eli Lilly, Janssen, Leopharma, Novartis, Pfizer, Sanofi and UCB. Evangelos J. Giamarellos‐Bourboulis received grants or contracts from AbbVie, Incyte, UCB, Novartis paid to the National and Kapodistrian University of Athens, Abbot Products Operations, bioMérieux, ENTEGRION, SOBI, Horizon EU grants ImmunoSep/EPIC‐CROWN‐2/POINT/HomiLung paid to the Hellenic Institute for the Study of Sepsis, received consulting fees from SOBI paid to the National and Kapodistrian University of Athens; and honoraria for lectures, presentations or manuscript writing from Abbot Products, BioTest, Operations, bioMérieux, SOBI, paid to the National and Kapodistrian University of Athens. Philippe Guillem has received honoraria from UCB, Novartis, Amgen and AbbVie. Ariela Hafner received consulting fees/honoraria from AbbVie and Novartis. John R. Ingram received a stipend as immediate past editor‐in‐chief of the British Journal of Dermatology and an authorship honorarium from UpToDate. He is a consultant for AbbVie, Boehringer Ingelheim, Cantargia, ChemoCentryx, Citryll, Engitix, Incyte, Insmed, Kymera Therapeutics, MoonLake, Novartis, UCB Pharma, UNION Therapeutics and Viela Bio. He is a co‐copyright holder of HiSQOL, Investigator Global Assessment and Patient Global Assessment instruments for HS, and his department receives income from copyright of the Dermatology Life Quality Index (DLQI) and related instruments. Niamh Kearney has received consulting fees, honoraria and travel grants from UCB, BMS, Janssen, AbbVie and Lilly. Natalia Kirsten has received honoraria and travel grants from AbbVie, Eli Lilly, Novartis, Leo Pharma, UCB, Janssen, Uluru Inc., Galderma, Pfizer, Celgene and Sanofi. Vesta Kucinskiene has received honoraria for presentations from Novartis, Janssen and travel grants from AbbVie and Novartis. Aikaterini I. Liakou has received advisory board fees from Novartis, UCB; lecture honoraria and support for attending meetings from Amgen, AbbVie, Boehringer Ingelheim, Novartis and UCB Pharma. She is sub‐investigator in clinical trials of AbbVie, Boehringer Ingelheim, Insmed, Novartis, Sanofi and UCB. Flavia Manzo Margiotta has received honoraria from AbbVie, Almirall, Canova, Eli Lilly, Leopharma, Pfizer and Sanofi. Antonio Martorell has received honoraria and/or travel grants and/or has served as an advisory board member for Novartis, AbbVie, Janssen Cilag, UCB, Lilly, LEO Pharma, L'Oréal, Sanofi, Boehringer Ingelheim, Almirall, Bristol Myers Squibb and Amgen. Additionally, AM has worked as a principal investigator in clinical trials supported by AbbVie, UCB, Janssen, Bristol Myers Squibb, Lilly, Galderma, Sanofi and Novartis. Łukasz Matusiak received consulting fees from AbbVie, Novartis, Leo Pharma, UCB, honoraria for lectures from AbbVie, Aristo Pharma, Leo Pharma, Medac, La Roche‐Posay, Almirall, UCB, Janssen, Novartis, Pierre‐Fabre and Pfizer; support for attending meetings from AbbVie and Novartis, participated on data safety monitoring boards or advisory boards for AbbVie, Leo Pharma, Novartis and UCB; and he has a leading role in the Polish Dermatology Society Guideline Committee. Alejandro Molina‐Leyva has received honoraria from AbbVie, Novartis, UCB and Almirall. Angelo Valerio Marzano reports consultancy/advisory boards disease‐relevant honoraria from AbbVie, Amgen, Boehringer Ingelheim, Bristol Myers Squibb, Incyte, Leopharma, Novartis, Pfizer, Sanofi and UCB. Francesca Prignano received consulting fees from AbbVie, Novartis, Eli‐Lilly, Almirall and Pfizer; received honoraria for lectures from AbbVie, Novartis, Eli‐Lilly, Almirall, Pfizer and Boehringer Ingelheim; support for attending meetings from Novartis and AbbVie; has participated on safety monitoring boards or advisory boards for AbbVie, Jannsen‐Cilag, Boehringer Ingelheim and Amgen; and has a leading or fiduciary role for SIDeMAST, EADV. Tadas Raudonis received grants or contracts from AbbVie for the Upadacitinib clinical trial, honoraria for lectures from AbbVie, Eli Lilly, Janssen, Novartis, support for attending meetings from AbbVie, Novartis, Janssen and is a board member of the Lithuanian Association of Dermatovenereology. Ditte Marie L. Saunte reports personal honoraria and grants from Galderma, Janssen, Leo Pharma, Pfizer, UCB, AbbVie, Sanofi and Novartis outside the submitted work. Thrasyvoulos Tzellos has received honoraria from UCB, Novartis, Almirall, BMS, AbbVie and MSD. Hans Christian Ring has received consulting fees from UCB, Novartis and Pierre Fabre; payment or honoraria for lectures from UCB, Novartis and Janssen; support for attending the EHSF 2025 from AbbVie and has participated in advisory boards for UCB and Novartis. Linnea Thorlacius is the Co‐copyright holder of HiSQOL© (Hidradenitis Suppurativa Quality of Life), HS‐IGA© (Hidradenitis Suppurativa Investigator Global Assessment), and HIDE© (Hidradenitis Suppurativa Drainage). Investigator for UCB, Novartis, Janssen and Incyte. Speaker fee from UCB. Hessel H van der Zee has received honoraria from UCB, Novartis, AbbVie, Incyte, Insmed and Sanofi. Kelsey van Straalen has received consulting fees from Novartis, honoraria for lectures from Novartis, UCB and Boehringer Ingelheim. Christos C. Zouboulis has received honoraria as a consultant for Almirall, Biogen, Boehringer Ingelheim, CLS Behring, Eli Lilly and Company, Estée Lauder, Idorsia, Incyte, Leo, L'Oréal, MSD, NAOS‐BIODERMA, Novartis, PPM, Sanofi, ShiRhom, Takeda, UCB and ZuraBio; and lecture honoraria from Almirall, Amgen, NAOS‐BIODERMA, Biogen, BMS, L'Oréal, Novartis, Pfizer and UCB. His departments have received grants from his participation as a clinical and research investigator for AstraZeneca, Boehringer Ingelheim, BMS, Brandenburg Medical School Theodor Fontane, EADV, European Union, German Federal Ministry of Education and Research, GSK, Incyte, InflaRx, MSD, Novartis, Relaxera, Sanofi and UCB. He is President of the EHSF e.V., president of the Deutsches Register Morbus Adamantiades‐Behçet e.V., board member of the International Society for Behçet's Disease, coordinator of the ALLOCATE Skin group of the ERN Skin and chair of the ARHS Task Force group of the EADV. He is Editor of the EADV News and co‐copyright holder of IHS4 on behalf of the EHSF e.V. The rest of the authors have no relevant conflicts of interest to declare.

## ETHICAL APPROVAL

Not applicable.

## ETHICS STATEMENT

Not applicable.

## Supporting information


Data S1.

## Data Availability

The data that support the findings of this study are available from the corresponding author upon reasonable request.

## References

[1] Zouboulis CC , Benhadou F , Byrd AS , Chandran NS , Giamarellos‐Bourboulis EJ , Fabbrocini G , et al. What causes hidradenitis suppurativa?—15 years after. Exp Dermatol. 2020;29(12):1154–1170. 33058306 10.1111/exd.14214

[2] Zouboulis CC , Nogueira da Costa A , Makrantonaki E , Hou XX , Almansouri D , Dudley JT , et al. Alterations in innate immunity and epithelial cell differentiation are the molecular pillars of hidradenitis suppurativa. J Eur Acad Dermatol Venereol. 2020;34(4):846–861. 31838778 10.1111/jdv.16147

[3] Zouboulis CC , Bechara FG , Fritz K , Goebeler M , Hetzer FH , Just E , et al. S2k guideline for the treatment of hidradenitis suppurativa/acne inversa (ICD‐10 code L73.2). Aktuelle Dermatol. 2024;50(1–2):30–83. 10.1111/ddg.15412 38770982

[4] Kimball AB , Okun MM , Williams DA , Gottlieb AB , Papp KA , Zouboulis CC , et al. Two phase 3 trials of Adalimumab for hidradenitis Suppurativa. N Engl J Med. 2016;375(5):422–434. 27518661 10.1056/NEJMoa1504370

[5] Rosi E , Fastame MT , Scandagli I , Di Cesare A , Ricceri F , Pimpinelli N , et al. Insights into the pathogenesis of HS and Therapeutical approaches. Biomedicines. 2021;9(9):1168. 34572354 10.3390/biomedicines9091168 PMC8467309

[6] Kimball AB , Jemec GBE , Alavi A , Reguiai Z , Gottlieb AB , Bechara FG , et al. Secukinumab in moderate‐to‐severe hidradenitis suppurativa (SUNSHINE and SUNRISE): week 16 and week 52 results of two identical, multicentre, randomised, placebo‐controlled, double‐blind phase 3 trials. Lancet. 2023;401(10378):747–761. 36746171 10.1016/S0140-6736(23)00022-3

[7] Kimball AB , Jemec GBE , Sayed CJ , Kirby JS , Prens E , Ingram JR , et al. Efficacy and safety of bimekizumab in patients with moderate‐to‐severe hidradenitis suppurativa (BE HEARD I and BE HEARD II): two 48‐week, randomised, double‐blind, placebo‐controlled, multicentre phase 3 trials. The Lancet. 2024;403(10443):2504–2519. 10.1016/S0140-6736(24)00101-6 38795716

[8] Kimball AB , Sobell JM , Zouboulis CC , Gu Y , Williams DA , Sundaram M , et al. HiSCR (hidradenitis Suppurativa clinical response): a novel clinical endpoint to evaluate therapeutic outcomes in patients with hidradenitis suppurativa from the placebo‐controlled portion of a phase 2 adalimumab study. J Eur Acad Dermatol Venereol. 2016;30(6):989–994. 26201313 10.1111/jdv.13216 PMC5034809

[9] Zouboulis CC , Hrvatin Stancic B , Abaitancei A , Guimarães MJ , Lobo IL , Massa AF , et al. The inter‐rater reliability of IHS4 corroborates its aptitude as primary outcome measurement instrument for large clinical studies in hidradenitis suppurativa. J Eur Acad Dermatol Venereol. 2024;38(2):e185–e187. 37728531 10.1111/jdv.19525

[10] van Straalen KR , Tzellos T , Alavi A , Benhadou F , Cuenca‐Barrales C , Daxhelet M , et al. External validation of the IHS4‐55 in a European antibiotic‐treated hidradenitis Suppurativa cohort. Dermatology. 2023;239(3):362–367. 36630943 10.1159/000528968 PMC10332479

[11] Tzellos T , van Straalen KR , Kyrgidis A , Alavi A , Goldfarb N , Gulliver W , et al. Development and validation of IHS4‐55, an IHS4 dichotomous outcome to assess treatment effect for hidradenitis suppurativa. J Eur Acad Dermatol Venereol. 2023;37(2):395–401. 36184889 10.1111/jdv.18632

[12] Zouboulis CC , Tzellos T , Kyrgidis A , Jemec GBE , Bechara FG , Giamarellos‐Bourboulis EJ , et al. Development and validation of the international hidradenitis Suppurativa severity score system (IHS4), a novel dynamic scoring system to assess HS severity. Br J Dermatol. 2017;177(5):1401–1409. 28636793 10.1111/bjd.15748

[13] Sartorius K , Emtestam L , Jemec GBE , Lapins J . Objective scoring of hidradenitis suppurativa reflecting the role of tobacco smoking and obesity. British Journal of Dermatology. 2009;161(4):831–839. 19438453 10.1111/j.1365-2133.2009.09198.x

[14] Mendonça CO , Griffiths CEM . Clindamycin and rifampicin combination therapy for hidradenitis suppurativa. Br J Dermatol. 2006;154(5):977–978. 16634904 10.1111/j.1365-2133.2006.07155.x

[15] Jemec GBE , Wendelboe P . Topical clindamycin versus systemic tetracycline in the treatment of hidradenitis suppurativa. J Am Acad Dermatol. 1998;39(6):971–974. 9843011 10.1016/s0190-9622(98)70272-5

[16] Van Der Zee HH , Boer J , Prens EP , Jemec GBE . The effect of combined treatment with oral clindamycin and oral rifampicin in patients with hidradenitis suppurativa. Dermatology. 2009;219(2):143–147. 19590174 10.1159/000228337

[17] Delaunay J , Villani AP , Guillem P , Tristan A , Boibieux A , Jullien D . Oral ofloxacin and clindamycin as an alternative to the classic rifampicin–clindamycin in hidradenitis suppurativa: retrospective analysis of 65 patients. Br J Dermatol. 2018;178(1):e15–e16. 28626900 10.1111/bjd.15739

[18] Albrecht J , Barbaric J , Nast A . Rifampicin alone may be enough: is it time to abandon the classic oral clindamycin–rifampicin combination for hidradenitis suppurativa? Br J Dermatol. 2019;180(4):949–950. 10.1111/bjd.17422 30430552

[19] Gener G , Canoui‐Poitrine F , Revuz JE , Faye O , Poli F , Gabison G , et al. Combination therapy with clindamycin and rifampicin for hidradenitis suppurativa: a series of 116 consecutive patients. Dermatology. 2009;219(2):148–154. 19590173 10.1159/000228334

[20] Yao Y , Jørgensen AHR , Ring HC , Thomsen SF . Effectiveness of clindamycin and rifampicin combination therapy in hidradenitis suppurativa: a 6‐month prospective study. Br J Dermatol. 2021;184(3):552–553. 33000461 10.1111/bjd.19578

[21] Dessinioti C , Zisimou C , Tzanetakou V , Stratigos A , Antoniou C . Oral clindamycin and rifampicin combination therapy for hidradenitis suppurativa: a prospective study and 1‐year follow‐up. Clin Exp Dermatol. 2016;41(8):852–857. 27753139 10.1111/ced.12933

[22] Bettoli V , Zauli S , Borghi A , Toni G , Minghetti S , Ricci M , et al. Oral clindamycin and rifampicin in the treatment of hidradenitis suppurativa‐acne inversa: a prospective study on 23 patients. J Eur Acad Dermatol Venereol. 2014;28(1):125–126. 23451831 10.1111/jdv.12127

[23] Marasca C , Masarà A , Annunziata MC , Bettoli V , Luciano MA , Fabbrocini G . Long‐term clinical safety of clindamycin and rifampicin combination for the treatment of hidradenitis suppurativa: a strategy to reduce side‐effects, improving patients' compliance. Br J Dermatol. 2019;180(4):949. 10.1111/bjd.17423 30430549

[24] Rosi E , Pescitelli L , Ricceri F , Di Cesare A , Novelli A , Pimpinelli N , et al. Clindamycin as unique antibiotic choice in hidradenitis Suppurativa. Dermatol Ther. 2019;32(2):e12792. 30515931 10.1111/dth.12792

[25] van Straalen KR , Tzellos T , Guillem P , Benhadou F , Cuenca‐Barrales C , Daxhelet M , et al. The efficacy and tolerability of tetracyclines and clindamycin plus rifampicin for the treatment of hidradenitis suppurativa; results of a prospective European cohort study. J Am Acad Dermatol. 2021;85(2):369–378. 33484766 10.1016/j.jaad.2020.12.089

[26] Zouboulis CC , Bechara FG , Fritz K , Kurzen H , Liakou AI , Marsch WC , et al. S1‐Leitlinie zur Therapie der Hidradenitis suppurativa/Acne inversa * (ICD‐10 Ziffer: L73.2). JDDG ‐ Journal of the German Society of Dermatology. 2012;10(SUPPL. 5):S1–S31. 10.1111/j.1610-0387.2012.08006.x 22925400

[27] Kirsten N , Frings V , Nikolakis GD , Presser D , Goebeler M , Zouboulis CC , et al. Epidemiology, patient quality of life, and treatment costs of hidradenitis suppurativa/acne inversa. Hautarzt. 2021;72(8):651–657. 34223939 10.1007/s00105-021-04851-z

[28] Khalsa A , Liu G , Kirby JS . Increased utilization of emergency department and inpatient care by patients with hidradenitis suppurativa. J Am Acad Dermatol. 2015;73(4):609–614. 26190241 10.1016/j.jaad.2015.06.053

[29] Melgosa Ramos FJ , García‐Ruiz R , Mateu Puchades A , Martorell A . Can we improve prognosis in hidradenitis Suppurativa? Identifying patients in the window of opportunity. Actas Dermosifiliogr. 2024;115(2):213–214. 10.1016/j.ad.2023.11.012 38048943

[30] Marzano AV , Genovese G , Casazza G , Moltrasio C , Dapavo P , Micali G , et al. Evidence for a “window of opportunity” in hidradenitis suppurativa treated with adalimumab: a retrospective, real‐life multicentre cohort study. Br J Dermatol. 2021;184(1):133–140. 32119111 10.1111/bjd.18983

[31] Haselgruber S , Fernández‐Crehuet‐Serrano P , Fernández‐Ballesteros MD , Padial‐Gómez A , Hernández‐Rodríguez JC , Ortiz‐Álvarez J , et al. Insights into the window of opportunity and outcome measures in patients with moderate to severe hidradenitis Suppurativa treated with Secukinumab: a real‐world study. Dermatol Ther (Heidelb). 2024;14(7):1875. 38896382 10.1007/s13555-024-01209-w PMC11264522

[32] Aggarwal R , Mahajan P , Pandiya S , Bajaj A , Verma SK , Yadav P , et al. Antibiotic resistance: a global crisis, problems and solutions. Crit Rev Microbiol. 2024;50(5):896–921. 38381581 10.1080/1040841X.2024.2313024

[33] Martorell A , Jfri A , Koster SBL , Gomez‐Palencia P , Solera M , Alfaro‐Rubio A , et al. Defining hidradenitis suppurativa phenotypes based on the elementary lesion pattern: results of a prospective study. J Eur Acad Dermatol Venereol. 2020;34(6):1309–1318. 10.1111/JDV.16183 31919904

[34] Van Der Zee HH , Jemec GBE . New insights into the diagnosis of hidradenitis suppurativa: clinical presentations and phenotypes. J Am Acad Dermatol. 2015;73:S23–S26. 10.1016/j.jaad.2015.07.047 26470610

[35] Nast A , Altenburg A , Augustin M , Boehncke WH , Härle P , Klaus J , et al. German S3‐guideline on the treatment of psoriasis vulgaris, adapted from EuroGuiDerm ‐ part 1: treatment goals and treatment recommendations. J Dtsch Dermatol Ges. 2021;19(6):934‐150. 10.1111/ddg.14508 34139083

[36] Nast A , Altenburg A , Augustin M , Boehncke WH , Härle P , Klaus J , et al. German S3‐guideline on the treatment of psoriasis vulgaris, adapted from EuroGuiDerm ‐ part 2: treatment monitoring and specific clinical or comorbid situations. J Dtsch Dermatol Ges. 2021;19(7):1092–1115. 10.1111/ddg.14507 34288477

[37] Mrowietz U , Augustin M . Using the upgrade criteria of the European psoriasis consensus is best practice care according to the people‐centred healthcare concept of the World Health Organization. Br J Dermatol. 2022;187(6):1007–1008. 36464934 10.1111/bjd.21827

[38] Frew JW , Jiang CS , Singh N , Grand D , Navrazhina K , Vaughan R , et al. Clinical response rates, placebo response rates, and significantly associated covariates are dependent on choice of outcome measure in hidradenitis suppurativa: a post hoc analysis of PIONEER 1 and 2 individual patient data. J Am Acad Dermatol. 2020;82(5):1150–1157. 31881294 10.1016/j.jaad.2019.12.044 PMC7167597

[39] Frew JW , Jiang CS , Singh N , Grand D , Navrazhina K , Vaughan R , et al. Dermal tunnels influence time to clinical response and family history influences time to loss of clinical response in patients with hidradenitis suppurativa treated with adalimumab. Clin Exp Dermatol. 2021;46(2):306–313. 10.1111/ced.14448 32931599

[40] Nikolakis G , Join‐Lambert O , Karagiannidis I , Guet‐Revillet H , Zouboulis CC , Nassif A . Bacteriology of hidradenitis suppurativa/acne inversa: a review. J Am Acad Dermatol. 2015;73(5):S12–S18. 26470608 10.1016/j.jaad.2015.07.041

[41] Nikolakis G , Liakou AI , Bonovas S , Seltmann H , Bonitsis N , Join‐Lambert O , et al. Bacterial colonization in hidradenitis suppurativa/acne inversa: a cross‐sectional study of 50 patients and review of the literature. Acta Derm Venereol. 2017;97(4):493–498. 27882387 10.2340/00015555-2591

[42] Kathju S , Lasko LA , Stoodley P . Considering hidradenitis suppurativa as a bacterial biofilm disease. FEMS Immunol Med Microbiol. 2012;65(2):385–389. 22353357 10.1111/j.1574-695X.2012.00946.x

[43] Ring HC , Bay L , Nilsson M , Kallenbach K , Miller IM , Saunte DM , et al. Bacterial biofilm in chronic lesions of hidradenitis suppurativa. Br J Dermatol. 2017;176(4):993–1000. 27564400 10.1111/bjd.15007

[44] Parsek MR , Singh PK . Bacterial biofilms: an emerging link to disease pathogenesis. Annu Rev Microbiol. 2003;57:677–701. 14527295 10.1146/annurev.micro.57.030502.090720

[45] Rosi E , Guerra P , Silvi G , Nunziati G , Scandagli I , Di Cesare A , et al. Consistency of bacterial triggers in the pathogenesis of hidradenitis Suppurativa. Vaccines (Basel). 2023;11(1):179. 36680023 10.3390/vaccines11010179 PMC9867521

[46] Zouboulis CC , Dessau SK , Hsiao J , Reguiai Z , Becherel P‐A , Kirby B , et al. 52756 Bimekizumab impact on draining tunnels in patients with moderate to severe hidradenitis suppurativa: pooled 48‐week data from BE HEARD I & II. J Am Acad Dermatol. 2024;91(3):AB144.

[47] Kokolakis G , Wolk K , Schneider‐Burrus S , Kalus S , Barbus S , Gomis‐Kleindienst S , et al. Delayed diagnosis of hidradenitis Suppurativa and its effect on patients and healthcare system. Dermatology. 2020;236(5):421–430. 32610312 10.1159/000508787 PMC7592906

[48] Fite C , Taieb C , Nassif A , Delage‐Toriel M , Cassius C , Skayem C , et al. Diagnostic wandering in hidradenitis suppurativa: a nationwide cohort study. J Eur Acad Dermatol Venereol. 2024;39(8):e668–e670. 39523774 10.1111/jdv.20425 PMC12291025

[49] Pasquale TR , Tan JS . Nonantimicrobial effects of antibacterial agents. Clin Infect Dis. 2005;40(1):127–135. 15614702 10.1086/426545

[50] Sensi P . History of the development of rifampin. Rev Infect Dis. 1983;5:S402–S406. 6635432 10.1093/clinids/5.supplement_3.s402

[51] Rifampin in chronic granulomatous disease. N Engl J Med. 1980;303(2):111. 10.1056/NEJM198007103030217 7383064

[52] Humbert P , Treffel P , Chapuis JF , Buchet S , Derancourt C , Agache P . The tetracyclines in dermatology. J Am Acad Dermatol. 1991;25(4):691–697. 1791227 10.1016/0190-9622(91)70255-z

[53] Webster GF , Toso SM , Hegemann L . Inhibition of a model of in vitro granuloma formation by Tetracyclines and ciprofloxacin: involvement of protein kinase C. Arch Dermatol. 1994;130(6):748–752. 8002645

[54] Brinkmeier T , Frosch PJ . Orale antibiotika mit antiinflammatorischer/immunmoduiatorischer wirkung für die therapie verschiedener dermatosen. Hautarzt. 2002;53(7):456–465. 12219268 10.1007/s00105-001-0334-4

[55] Zouboulis CC , Bechara FG , Fritz K , Goebeler M , Hetzer FH , Just E , et al. S2k guideline for the treatment of hidradenitis suppurativa / acne inversa – short version. JDDG J Dtsch Dermatol Ges. 2024;22:868–889. 38770982 10.1111/ddg.15412

[56] Fischer AH , Haskin A , Okoye GA . Patterns of antimicrobial resistance in lesions of hidradenitis suppurativa. J Am Acad Dermatol. 2017;76(2):309–313.e2. 27742173 10.1016/j.jaad.2016.08.001

[57] Dréno B . Bacteriological resistance in acne: a call to action. Eur J Dermatol. 2016;26(2):127–132. 26711531 10.1684/ejd.2015.2685

