# Supplementary material for: Towards the development of upgrade criteria for the treatment of hidradenitis suppurativa with biologics
Source: J Eur Acad Dermatol Venereol. 2025 Nov 13;40(4):658–69. doi: 10.1111/jdv.70118 (PMC13014438; doi:10.1111/jdv.70118)
Supplement: Supplementary file 1 — Data S1. [file JDV-40-658-s001.docx]

**Article type:** Original article

**Title:** Towards the development of upgrade criteria for the treatment of hidradenitis suppurativa with biologics.

**Running title:** HS: upgrade criteria for biologics

Georgios Nikolakis^1,2^, Erkan Alpsoy^2,3^, Monika Arenbergerova^4^, Falk G. Bechara^2,5^, Farida Benhadou^2,6^, Joana Cabete^2,7^, Raffaele Dante Caposiena Caro^2,8^, Giovanni Damiani^2,9,10^, Maia Delage Toriel^2,11^, Veronique Del Marmol^2,12^, Valentina Dini^2,13^, Evangelos J. Giamarellos-Bourboulis^2,14^, Katalin Glasenhardt^2,15^, Philippe Guillem^2,16^, Ariela Hafner^2,17^, Evgenya Hristakieva^2,18,19^, John R. Ingram^2,20^, Vaiva Jarienè^2,21^, Gregor B.E. Jemec^2,22,23^, Niamh Kearney^2,24,^, Brian Kirby^2,25,26^, Natalia Kirsten^27^, Piotr K. Krajewski^2,28^, Vesta Kucinskiene^2,21^, Aikaterini I. Liakou^2,29^, Flavia Manzo Margiotta^2,14,30^, Angelo V. Marzano^2,31,32^, Antonio Martorell^2,33^, Lukasz Matusiak^2,34^, Dillon Mintoff^2,35^, Alejandro Molina Leyva^2,36,37^, Francesca Prignano^2,38^, Tadas Raudonis^2,39^, Hans Christian Ring^40^, Marco Romanelli^2,14,30^, Sahin Samed^2,41^, Ditte ML Saunte^2,22,42^, Linnea Thorlacius^41,43^, Thrasyvoulos Tzellos^2,44^, Hessel H. Van Der Zee^2,45^, Kelsey Van Straalen^2,45^, Christos C. Zouboulis^1,2^

1. Departments of Dermatology, Venereology, Allergology and Immunology, Städtisches Klinikum Dessau, Brandenburg Medical School Theodor Fontane and Faculty of Health Sciences Brandenburg, Dessau, Germany
2. European Hidradenitis Suppurativa Foundation e.V., Dessau, Germany
3. Department of Dermatology and Venereology, Akdeniz University, Antalya, Türkiye
4. Department of Dermatovenerology, Third Faculty of Medicine, Charles University and University Hospital Kralovske Vinohrady, Prague, Czech Republic
5. International Centre for Hidradenitis Suppurativa/Acne Inversa (ICH), Department of Dermatology, Venereology and Allergology, Ruhr-University Bochum, Bochum, Germany
6. Department of Dermatology, Hôpitaux Universitaires de Bruxelles (HUB), Université libre de Bruxelles, Brussels, Belgium
7. Department of Dermatology and Venereology, Hospital de Santo António dos Capuchos - ULS de São José, Lisbon, Portugal
8. Dermatology Clinic, Maggiore Hospital, University of Trieste, Trieste, Italy.
9. Department of Biomedical, Surgical and Dental Sciences, University of Milan, Milan, Italy
10. Italian Center of Precision Medicine and Chronic Inflammation, University of Milan, Milan, Italy
11. L’Oreal et Institut Pasteur, Paris, France
12. Department of Dermatology, Hôpital Erasme - Hôpitaux universitaires de Bruxelles (HUB), Brussels, Belgium
13. Dermatology Unit, Department of Clinical and Experimental Medicine Ospedale Santa Chiara, Pisa, Italy
14. 4^th^ Department of Internal Medicine, National and Kapodistrian University of Athens, Medical School, Athens, Greece
15. Department of Dermatology and Allergology, University of Szeged, Szeged, Hungary
16. Clinique du Val d'Ouest, Service de Chirurgie, Ecully, France
17. Department of Dermatology, Tel Aviv Sourasky Medical Center, Tel Aviv, Israel
18. Clinic of Dermatology and Venereology, UMHAT "Prof. Dr. Stoyan Kirkovich" AD, Stara Zagora, Bulgaria
19. Section of Dermatovenereology, Faculty of Medicine, Trakia University, Stara Zagora, Bulgaria
20. Division of Infection and Immunity, Cardiff University, Cardiff, UK
21. Department of Skin and Venereal Diseases, Lithuanian University of Health Sciences (LSMU); Hospital of LSMU Kauno Klinikos, Kaunas, Lithuania
22. Department of Allergy and Dermatology, Herlev & Gentofte University Hospital, Gentofte, Denmark.
23. Department of Clinical Medicine, Faculty of Health and Medical Sciences, University of Copenhagen, Denmark
24. Department of Dermatology, Our Lady of Lourdes Hospital Drogheda, Ireland
25. School of Medicine, University College Dublin, Ireland
26. Department of Dermatology, St. Vincent's University Hospital Dublin, Ireland
27. Department of Dermatology and Allergy, University Hospital, LMU Munich, Munich, Germany
28. University Centre of General Dermatology and Oncodermatology, Wroclaw Medical University, Wroclaw, Poland
29. 1^st^ Department of Dermatology-Venereology, "Andreas Sygros" Hospital, Medical School, National and Kapodistrian University of Athens, Athens, Greece
30. Health Science Interdisciplinary Center, Sant'Anna School of Advanced Studies, Pisa, Italy
31. Dermatology Unit, Fondazione IRCCS Ca' Granda Ospedale Maggiore Policlinico, Milan, Italy
32. Department of Pathophysiology and Transplantation, Università degli Studi di Milano, Milan, Italy
33. Department of Dermatology, Hospital de Manises, Valencia, Spain
34. Faculty of Medicine, Wroclaw University of Science and Technology, Wroclaw, Poland
35. Department of Dermatology, Mater Dei Hospital, Msida, Malta
36. Hospital Universitario Virgen de las Nieves, Servicio de Dermatología-Ibs.Granada, Granada, Spain
37. Department of Dermatology. University of Granada, Granada, Spain.
38. Department of Health Science, Dermatology Section, University of Florence, Florence, Italy
39. Clinic of Infectious Disease and Dermatovenereology, Institute of Clinical Medicine, Faculty of Medicine, Vilnius University, Vilnius, Lithuania
40. Department of Dermatology, Zealand University Hospital, Roskilde, Denmark
41. Department of Dermatology, Gazi University Faculty of Medicine, Ankara, Turkey
42. Afdelingen for Hud- og Kønssygdomme, Aarhus Universitetshospital, Aarhus, Denmark
43. Section for Biostatistics and Evidence-Based Research, the Parker Institute, Bispebjerg and Frederiksberg Hospital, Copenhagen, Denmark
44. Department of Dermatology, Nordland Hospital Trust, Bodø, Norway
45. Department of Dermatology, Erasmus medical center, Rotterdam, the Netherlands

**Supplemental material**

**Tables**

Table 1

| Descriptives | | |
| --- | --- | --- |
|  | **Group** | **Age** |
| **N** | **Experts** | 23 |
|  | **Future opinion leaders** | 20 |
| **Mean** | **Experts** | 55.4 |
|  | **Future opinion leaders** | 36.5 |
| **Standard deviation** | **Experts** | 7.80 |
|  | **Future opinion leaders** | 4.88 |
| **Shapiro-Wilk W** | **Experts** | 0.943 |
|  | **Future opinion leaders** | 0.968 |
| **Shapiro-Wilk p** | **Experts** | 0.211 |
|  | **Future opinion leaders** | 0.704 |

**Figures**

Figure 1


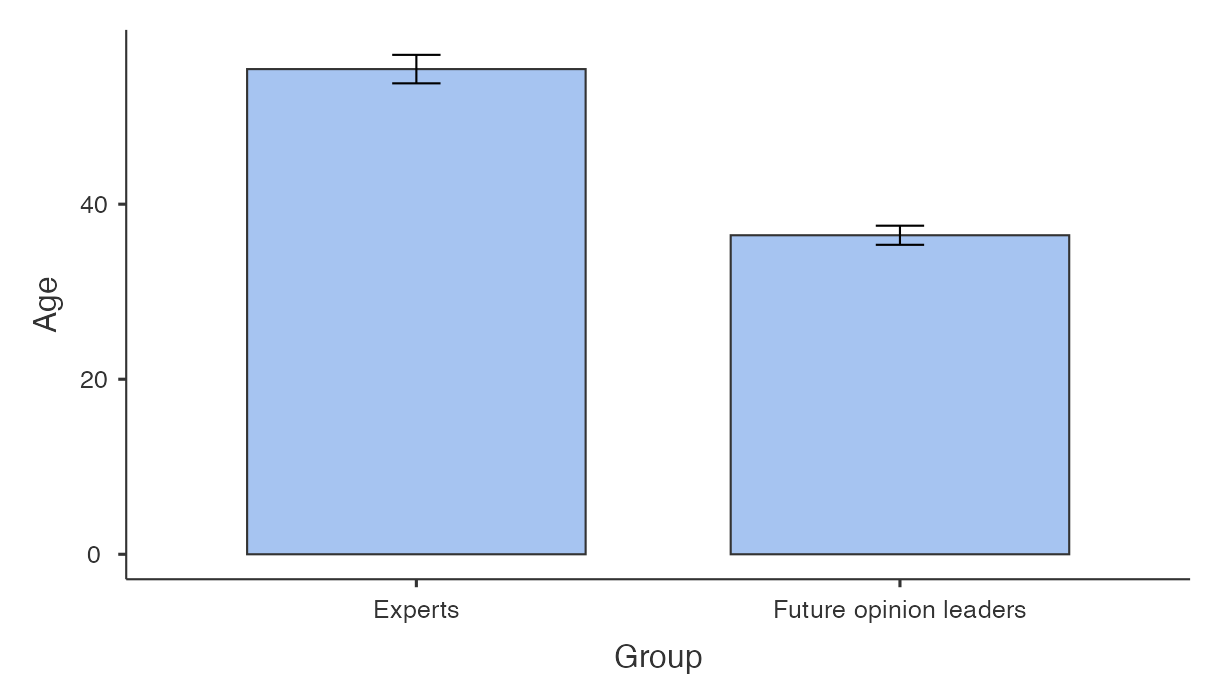


**Legends**

**Table 1:** Demographic characteristics and normality test results for age in expert and future opinion leader groups.

N = Number of participants in each group; Mean = Mean age; SD = Standard deviation; Shapiro-Wilk W = Test statistic for normality; Shapiro-Wilk p = p-value for normality. Data show that both groups' ages are approximately normally distributed (Shapiro-Wilk p >0.05 for both groups)

**Figure 1:** Age distribution among expert and future opinion leader subgroups.
